# Role of the Vasa Vasorum and Vascular Resident Stem Cells in Atherosclerosis

**DOI:** 10.1155/2014/701571

**Published:** 2014-03-05

**Authors:** Jun-ichi Kawabe, Naoyuki Hasebe

**Affiliations:** Department of Cardiovascular Regeneration and Innovation and Department of Medicine, Division of Cardiovascular, Respiratory and Neurology, Asahikawa Medical University, 2-1-1-1 Midorigaoka-higashi, Asahikawa 078-8510, Japan

## Abstract

Atherosclerosis is considered an “inside-out” response, that begins with the dysfunction of intimal endothelial cells and leads to neointimal plaque formation. The adventitia of large blood vessels has been recognized as an active part of the vessel wall that is involved in the process of atherosclerosis. There are characteristic changes in the adventitial vasa vasorum that are associated with the development of atheromatous plaques. However, whether vasa vasorum plays a causative or merely reactive role in the atherosclerotic process is not completely clear. Recent studies report that the vascular wall contains a number of stem/progenitor cells that may contribute to vascular remodeling. Microvessels serve as the vascular niche that maintains the resident stem/progenitor cells of the tissue. Therefore, the vasa vasorum may contribute to vascular remodeling through not only its conventional function as a blood conducting tube, but also its new conceptual function as a stem cell reservoir. This brief review highlights the recent advances contributing to our understanding of the role of the adventitial vasa vasorum in the atherosclerosis and discusses new concept that involves vascular-resident factors, the vasa vasorum and its associated vascular-resident stem cells, in the atherosclerotic process.

## 1. Introduction

Atherosclerosis, a chronic progressive inflammatory disease of the arterial wall, has traditionally been considered an “inside-out” response in which injury to intimal endothelial cells initiates the adhesion/invasion of inflammatory cells in the subendothelial space. Subsequently atherosclerotic plaques grow by the accumulation of inflammatory cells and lipid substances and the proliferation of vascular smooth muscle cells [1, 2]. Clinical evidence indicates that instability rather than plaque size affects the prognosis of cardiovascular diseases [3, 4]. However, the underlying mechanism driving the conversion of an asymptomatic stable atheroma to a lesion vulnerable plaque is not fully explained by the “inside-out” theory.

The adventitia is no longer viewed as a passive support structure in large vessels. The vascular adventitia, which harbors a wide variety of components such as fibroblasts, inflammatory cells, stem/progenitor cells, and vasa vasorum, can act as a biological central processing unit in vessel wall function. Recent emerging evidence proposes a new paradigm regarding the sites/direction of the atherosclerotic process, the “outside-in” hypothesis. Under this paradigm, vascular inflammation is initiated in the adventitia and progresses toward the intima [5, 6]. The vasa vasorum is an adventitial microvascular network that supplies oxygen and nutrients to the blood vessel walls. Numerous studies report that changes in vasa vasorum characteristics are closely associated with the development of atheromatous plaques. Consequently, it is proposed that the vasa vasorum appears to play key role in the “outside-in” model of atherosclerosis [5–7]. However, whether the vasa vasorum plays a causative or reactive role in the atherosclerotic process is not clearly understood.

A number of studies have demonstrated that the vascular walls act as a perivascular niche for stem/progenitor cells that contribute to vascular repair, fibrosis, and atherosclerosis (Table 1) [8, 9]. In particular, stem cells that associated with microvessels have been identified. Pericytes, also known as vascular mural cells, surround endothelial cells in capillaries and microvessels [10, 11]. Several recent studies demonstrated that a subpopulation of pericytes is multipotent. Multipotent pericytes can differentiate into mesodermal and ectodermal cell lineage, including smooth muscle cells, osteoblasts, adipocytes, and skeletal muscle cells [12–16]. Endothelial progenitor cells (EPCs) also exist within populations of tissue-resident endothelial cells [17]. Recently, an endothelial cell-like stem cell population has been identified as a side population of CD31+ cells that are located at the inner surface of preexisting microvessels and macrovessels [18]. Therefore, the vasa vasorum can contribute to vascular remodeling through not only its conventional function as a blood conduit tube, but also its newly proposed function as a stem cell reservoir. This brief review highlights recent advances in our understanding of the role of the adventitial vasa vasorum and its associated vascular stem cells in atherosclerosis and discusses progress toward an integrated view of adventitial function in atherosclerotic plaques.

## 2. Vasa Vasorum and Atherosclerotic Plaques

The vasa vasorum is a microvascular network that supplies oxygen and nutrients to the walls of large vessel. These conduits consist of a lumen lined by endothelial cells that are surrounded by pericytes or smooth muscle cells. Recent technological advances in image analysis have revealed that the enhanced vascularization in plaques is closely associated with the prognosis of acute arterial occlusion [6, 27]. Because of the histological characteristics of the vasa vasorum in plaque, it has been proposed that neovascularization of the vasa vasorum plays a role in the progression and associated complications of atherosclerotic plaques. The vasa vasorum is primarily located in the adventitial layer of large vessel and extends into atherosclerotic plaques.

In atherosclerotic plaques, the vasa vasorum is considered immature, a characteristic that leads to the microvascular leakage that is responsible for plaque hemorrhage. Because of its high permeability, the vasa vasorum also serves as a conduit for the delivery of inflammatory cells into the plaques. Plaque hemorrhage and inflammatory cell delivery are the key mechanisms underlying the persistence of chronic vascular inflammation and the rapid expansion or rupture of atherosclerotic plaques [28, 29].

### 2.1. The Association between the Vasa Vasorum and Neointimal Thickening

Experimental studies using atherosclerotic models, such as apolipoprotein E- (ApoE-) deficient mice, clearly demonstrate a correlation between vasa vasorum neovascularization and plaque progression [30–32]. A study aimed at visualizing and quantifying the three-dimensional spatial patterns of the vasa vasorum in normal and balloon-injured porcine coronary arteries demonstrated that the amount of adventitial neovascularization is proportional to the degree of injured arterial stenosis [33]. Tanaka et al. demonstrated that angiogenesis in the adventitia, induced by the local administration of the angiogenic factor bFGF, promoted the growth of atherosclerotic plaques in ApoE-deficient mice, supporting the notion that vasa vasorum formation plays a crucial role in the pathogenesis of atherosclerosis [32]. A major factor that induces pathological angiogenesis is the accumulation of inflammatory cells within plaques, which causes oxidative stress. The overexpression of a critical component of NAD(P)H oxidase, p22-phox, in the smooth muscle cells of transgenic mice causes oxidative stress in carotid lesions and triggers an *in vivo *angiogenic switch associated with experimental plaque progression and angiogenesis [30].

### 2.2. Does Expansion of the Vasa Vasorum Cause or React to Neointimal Thickening?

The findings highlighted above are consistent with an emerging concept suggesting that the expansion of the vasa vasorum causes the progression of atherosclerotic plaques; however, it is still controversial whether the vasa vasorum plays a causative or reactive role in the atherosclerotic process. In some cases, even a low density of vasa vasorum induces neointimal thickening. Khurana et al. reported that the application of the angiogenesis stimulator VEGF to injured rat arterial walls results in, but does not initiate, a marked increase in neointimal thickening [34]. In crossbred swine fed a high cholesterol diet, low-density vasa vasorum territories within the coronary vessel wall became susceptible to hypoxia, oxidative stress, and microinflammation, potential triggers of early atherogenesis [35].

In atherosclerotic plaques, neovascularization is the primary compensatory response to hypoxia and inflammatory conditions. Neointimal thickening causes ischemia, which strongly induces angiogenesis. Although the expansion of the vasa vasorum in response to neointimal thickening should improve intraplaque ischemia, it does not. Recently, Rademakers et al. investigated the vasa vasorum in plaques of atherosclerotic carotid arteries from aged ApoE-deficient mice by performing *in vivo* functional imaging using multiphoton laser-scanning microscopy. Interestingly, the enhanced plaque-associated immature vasa vasorum not only showed increased permeability, leukocyte adhesion, and intraplaque hemorrhage, but also showed reduced blood flow within the plaques [36]. In accordance with this finding, hyperglycemia altered the structure, but not the density of the vasa vasorum, and accelerated atherosclerosis [37]. In normoglycemic ApoE-deficient mice, atherogenesis is associated with vasa vasorum expansion, likely corresponding to the increasing blood supply demands of the thickening artery wall. By contrast, in the hyperglycemic group, there was no significant neovascularization of the vasa vasorum despite the fact that the lesions were significantly larger [37]. Therefore, it should be noted that not only the density, but also the structural features of the vasa vasorum affect atherogenesis, and the role of the adventitial vasa vasorum may vary depending on time elapsed after vascular injury.

## 3. Vasa Vasorum and Vascular-Resident Stem Cells

### 3.1. Vascular-Resident Stem Cells and Atherosclerosis

The contribution of vascular-resident stem/progenitor cells to atherosclerosis progression has been confirmed in recent studies utilizing animal models of atherosclerosis with vascular injury. The vascular-resident stem cells are capable of differentiating into myofibroblasts that subsequently migrate to the intima and contribute to the development of neointimal hyperplasia [8, 9]. Adventitial Sca1+ cells carrying a *lacZ* reporter gene were transferred to the adventitial side of vein grafts in ApoE-deficient mice. *β*-gal-positive transplanted cells were found in atherosclerotic lesions in the intima, and these cells enhanced the development of these lesions. Consequently, the authors proposed that a large population of vascular progenitor cells residing in the adventitia can differentiate into the vascular smooth muscle cells that contribute to atherosclerosis [19]. Human autopsies have demonstrated the presence of CD34+Sca1+CD133− cells within neointimal lesions and the adventitia of atherosclerotic plaques, which may be a source of endothelial and vascular smooth muscle cells that form atherosclerotic lesions [38].

### 3.2. The Contribution of the Vasa Vasorum as a Stem Cell Reservoir in Plaque Formation

In the adventitia, multipotent pericytes and endothelial progenitor cells exist as structural cells of the vasa vasorum. Capillary microvessels also provide a vascular niche to house perivascular stem cells [39]. Therefore, the adventitial vasa vasorum might serve as a major reservoir for vascular-resident stem cells. An expanded vasa vasorum may contribute to vascular remodeling by serving as a reservoir for vascular stem cells and a conduit for not only the delivery of inflammatory cells, but also the circulation of stem cells and resident stem cells in plaques [40–42].

**Figure 1:**
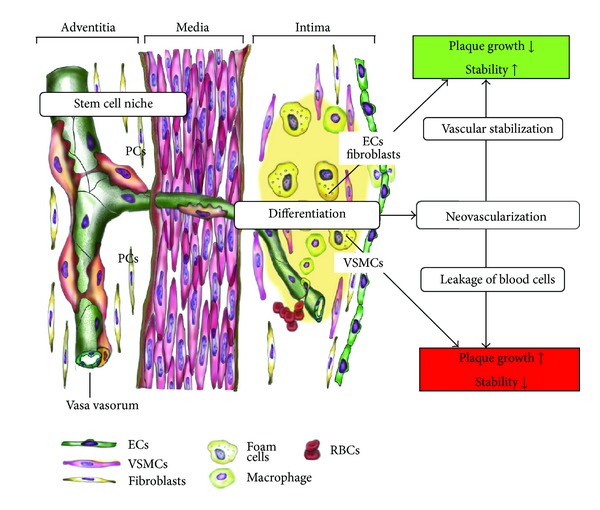
Role of the vasa vasorum in atherosclerosis. In atherosclerotic plaque, the vasa vasorum leads to the microvascular leakage that is responsible for hemorrhage and accumulation of inflammatory cells within plaque. Vasa vasorum also serves as the vascular niche for the vascular-resident stem cells (VSCs), including multipotent pericytes and endothelial progenitor cells. Vasa vasorum acts not only as the blood conduit tube but also as a stem cell reservoir to supply VSCs into the intima. VSCs can differentiate into several cells, such as vascular smooth muscle cells (VSMCs), endothelial cells (ECs), and fibroblasts, and can contribute to the atherosclerotic remodeling. Some of VSCs act as pericytes (PCs) to stabilize the vasculature, which attenuate the leakage of blood cells within plaques.

Previously, Diaz-Flores et al. reported that in rat femoral arteries that had the adventitial layers removed, the pericytes and endothelial cells of adventitial growing microvessels served as a source of myointimal cells at the intimal thickening and endothelium at the luminal surface, respectively [43]. Recent evidence suggests that the vasa vasorum-associated stem cells affect the prognosis of atherosclerosis. Using a mouse vein graft model, Chen et al. examined the effect of vasa vasorum-associated progenitor cells on atherosclerosis [44]. In ApoE-deficient mice, transplantation of Sca1^+^ cells that were in close proximity to the vasa vasorum to the outer layer of vein grafts enhanced atherosclerosis, contributing approximately 30% of the neointimal smooth muscle cells. Recently, Tigges et al. reported that adventitial multipotent pericytes participate in the restenotic response in mice with femoral arterial injuries [22]. These multipotent pericytes are increased in adventitia in response to vascular injury and contribute to restenosis in injured arteries. Adventitial pericytes have mesenchymal stem cell-like features and are potentially an important cellular source that contributes to intimal hyperplasia in rat aortic allograft models with transplantation-derived arteriosclerosis [45]. Collectively, these findings suggest that the expansion of the vasa vasorum contributes to a pool of vascular stem cells, including multipotent pericytes, and participates in the atherosclerotic process, in part, by supplying plaque-forming cells, includingsmooth muscle cells (Figure 1).

It is well documented that vascular stem cells migrate to the intimal sites and differentiate into myofibroblasts, contributing to neointimal thickening. When Sca1^+^ cells are transplanted to the adventitial side of vein grafts in ApoE-deficient mice, the cells migrate into the intima and differentiate into smooth muscle cells [19]. Tigerstedt et al. examined vascular cell kinetics in response to intimal injury *ex vivo* [46]. There is an influx of adventitial precursor cells in the intimal layer that occurs after rat aortic denudation injury. Cell migration was found to contribute to neointimal hyperplasia more than cell proliferation [46]; however, it is unclear how these stem cells migrate through the vascular walls. Díaz-Flores jr et al. investigated the vasa vasorum as a source of supplementary cells during intimal thickening by tracing labeled cells within vascular walls. This study provides evidence that adventitial microvascularization contributes to the delivery of supplementary population of fibroblast-like and myointimal cells into the neointima [47].

### 3.3. The Role of Vascular-Resident Stem Cells in Neovascularization

It is well documented that vascular stem cells have potent angiogenic effects through the paracrine effect and/or the differentiation into endothelial cells or pericytes (Table 1). Therefore, vascular stem cells may contribute to the growth of the vasa vasorum within atherosclerotic lesions.In normal human arteries, vascular progenitor-committed nestin+ cells are located in small-sized vasa vasorum. This could represent a valid evidence for the vasculogenic niche and potentially represents the main source for neovasculogenesis during atherosclerosis [48].As discussed previously, plaque neovessels are characterized as thin-walled with less investment by pericytes and are often of larger caliber than normal capillaries. This fragile structure could be regarded as sufficient in itself to render these vessels prone to the delivery of inflammatory cells, hemorrhage, and reduced blood flow. Pericytes play an important role in the regulation of vascular contractility and support their maturation and stability that fragile blood vessels become firm to suppress the leakage of the blood cells or hemorrhage [10, 49]. In addition to their potent regenerative activity, multipotent pericytes also play a role in vascular stabilization by structurally and functionally interacting with endothelial cells (Figure 1). Coculture of CD34+ multipotent pericytes with endothelial cells on Matrigel leads to the cooperative assembly of an endothelial network with enhanced stability [15]. Vascular-resident CD44+ multipotent stem cells give rise to pericytes, smooth muscle cells, and contribute to the vessel maturation [50]. To date, it is unclear whether angiogenesis of vascular-resident stem cells acts beneficially or detrimentally in the atherosclerotic pathogenesis. This may be changed depending on the kinds of stem cells and differentiated cells, that is, endothelial cells or pericytes, and the environmental conditions.

## 4. Antiatherosclerotic Therapy Based on Vasa Vasorum Biology

### 4.1. Antiangiogenesis Therapy

Antiatherosclerotic therapeutic strategies have been proposed based on findings describing the biology of the vasa vasorum in atherosclerotic plaques [5, 51]. Because a major determinant of plaque vulnerability and progression is the leakage of red blood cells from the vasa vasorum, the targeted inhibition of plaque angiogenesis may constitute a valuable therapeutic approach toward plaque stabilization and regression.

Moulton et al. reported that blocking vasa vasorum angiogenesis with angiostatin reduces the accumulation of macrophages in plaques and around the vasa vasorum and reduces the progression of atherosclerosis [52]. They propose that the inhibition of plaque angiogenesis and the secondary reduction of macrophages may have beneficial effects on plaque stability. Antiangiogenic PAI-1_23_, a truncated isoform of plasminogen activator inhibitor-1 promotes vasa vasorum regression and reduces atherosclerotic plaques in hypercholesterolemic mice through a plasmin-dependent mechanism [53, 54].

It is widely recognized that cholesterol-lowering statin drugs have potent antiatherosclerotic activity [55]. In addition to their cholesterol lowering effect, statins have pleiotropic pro- and antiangiogenic properties [56]. There is abundant evidence from both animal and human studies examining the effects of statins on angiogenesis in ischemic heart disease and stroke, but statins also have a potent antiangiogenic effect on atherosclerotic neovasculature. Independent of its cholesterol-lowering effects, simvastatin attenuated vasa vasorum neovascularization in a pig model fed a high cholesterol diet [57]. Similarly, independent of lowering cholesterol levels, atorvastatin significantly inhibited the development of adventitial vasa vasorum and the progression of atherosclerosis in a rabbit model of atherosclerosis [58].

### 4.2. Vascular Normalization Therapy

In addition to the density/expansion of the vasa vasorum, its structural and functional impairment play crucial roles in atherosclerotic plaque development. Fragile neovessels are formed within plaques, reducing perfusion flow regardless of the expansion of intraplaque vasa vasorum that contributes to plaque growth [36]. Therefore, the mere reduction of vasa vasorum density may decrease oxygenation and increase oxidative stress, initiating cascades of inflammation and intimal proliferation. Alternatively, the deletion of microvessels results in the depletion of the tissue-specific stem cell niche that subsequently becomes prematurely exhausted and unable to maintain organ function [59, 60]. Therefore, normalization of the impaired vasa vasorum would be an attractive therapeutic strategy rather than mere antiangiogenesis therapy. Although analytical techniques to image the vasa vasorum have advanced, histological methods to specifically visualize the vasa vasorum are limited. Therefore, experiments aimed at investigating vascular maturation/stabilization in the vasa vasorum, particularly in pathological settings, are limited.

The intimate interaction between pericytes and endothelial cells tightly correlates with vascular growth, maturation/stabilization, and remodeling of vessels [61]. Several external factors also may affect this interaction to regulate vascular maturation [62]. Recently, we developed an *in vivo* angiogenesis assay using collagen-coated tubes (CCTs) to observe the vasa vasorum of the injured mouse femoral artery. Using this novel angiogenesis assay, we found that nerve growth factors (NGF) had potent angiogenic effects on the microvessels around the injured artery and, more specifically, induced the maturation/stabilization of microvessels and the regeneration of perivascular nerves [63]. Lastly, we propose new strategies for the normalization of vasa vasorum by peripheral nerve innervation.

## 5. Conclusion

In this review, we discussed the effect of the vasa vasorum on the progression of atherosclerotic plaques with respect to its function not only as a conduit structure that delivers blood components, but also as a stem cell reservoir. A clearer understanding of adventitial vasa vasorum biology would provide insight that would lead to a better understanding of atherosclerotic pathogenesis and improved therapeutic strategies to combat atherosclerotic diseases. Pharmacological inhibition of angiogenesis in atherosclerotic plaques reportedly inhibits lesion progression in animal models. However, it is important to consider that the vasa vasorum acts as either a causative or responsive factor in neointimal formation depending on the atherosclerotic stage. Prior to designing clinical studies aimed at regulating angiogenesis in atherosclerotic diseases, the stage-dependent role of the vasa vasorum in atherosclerotic plaque development should be fully elucidated.

## Figures and Tables

**Table 1:** Resident progenitor cells within the vasculature.

Location	Name	Selection method	Markers	Comments	Reference
Adventitia	Vascular progenitor cells	**Sca1** positive cells from cells outgrown from mouse thoracic aorta adventitia	Sca1+	Differentiate into **SMCs**, contributing to the formation of hyperplasia of ApoE-deficient atherosclerotic lesions	Hu et al., 2004 [19]
	Vascular progenitor cells	**CD34+ CD31−** cells from human adult saphenous vein	CD34+	**MSC-** and **NSC-**like differentiation potential, but no endodermic differentiation was detected *at the clonal level *	Campagnolo et al., 2010 [20]
	(Saphenous vein-derived progenitor cells; **SVPs**)		After culture in the presence of serum, CD34 were subsided, and the following markers were increased:		
		(CD34+ cKit+ cells were located at perivascular sites of the **vasa vasorum**)	CD29+, CD44+, CD105+, SOX2+, Nestin+, NG2+ (CD146−)	Act as **PCs**, formed capillary structures (attached to EC-tubes), and improve ischemic hind limb through forming capillaries	
	Adventitial stem cells	**CD34+ CD146− cells** from human stromal adipose tissue around vascular adventitia	CD34+	CD34+ CD146− cells display **MSC-**like features *at the clonal level *	Corselli et al., 2012 [21]
			CD31−, CD146−, and CD45−		
				These cells acquired a **PCs-**like phenotype (NG2+, CD146+, etc.) in the presence of angiopoietin 2	
	Adventitial pericyte progenitor cells	**NG2+ CD146+cells** from vascular adventitia	NG2+, CD146+, PDGFR+	**MSC-like PCs** contribute to restenosis following arterial injury	Tigges et al., 2013 [22]
			Some of these cells also express CD29+, CD90+		
				NG2/CD146+ cells were increased in the adventitia of the injured vasculature	

Vasculogenic zone	Vascular wall resident **EPCs**	**CD34+ CD105−** cells from human internal thoracic artery	CD34+, KDR (VEGFR2)+, Tie2+	Differentiate into mature **ECs** forming new vessels and **hematopoietic cells** including macrophages	Zengin et al., 2006 [23]
			CD105−, CD144−		
		(CD34+ cells located at the “**vasculogenic zone**")			
	Angiogenic MSCs	Adherent culture condition (cells were isolated from human thoracic artery, and select the adherent cells forming colony )	MSC markers (CD44+, CD90+, CD105+, etc.)	Differentiate into **ECs** in the presence of VEGF, and form capillary-like structure	Pasquinelli et al., 2007 [24]
			CD45−, CD146−, vWF−	(These cells were heterogenous)	
		(CD34+ cKit+ cells were enriched in the **vasculogenic zone**)		It is uncertain whether the isolated cells are equivalent of CD34− stained cells within thoracic aorta	

Media	Side population-progenitor cells	Side population of cells from the tunica media of mouse aorta	Sca1+, cKit(dim),	Differentiate into **ECs** and **SMCs** to form vascular-like branching structures on Matrigel	Sainz et al., 2006 [25]
			CD34−, lineage negative	(These cells were heterogenous)	
	Multipotent vascular stem cells (**MVSCs**)	**SM-MHC (smooth muscle myosin heavy chain)—negative** cells from media of carotid artery of rats.	SM-MHC(−), Sox1+, Nestin+	**MSC-** and **NSC-**like multipotency at the clonal level	Tang et al., 2012 [26]
			CD146−, CD34−, CD31−		
				Differentiate into **SMCs** and **chondrogenic cells**, contributing to vascular remodeling and neointimal hyperplasia	

EPCs: endothelial progenitor cells; MSCs: mesenchymal stem cells; NSCs: neuronal stem cells; ECs: endothelial cells; SMCs: smooth muscle cells; PCs: pericytes. Vasculogenic zone: the border between the media and adventitial layer.

Stem cell/hematopoietic markers: CD34, Sca1, and cKit.

MSC markers: CD29, CD44, CD90, and CD105.

EC markers: CD31, vWF, and VEGFR.
